# Activity-Friendly Built Environments in a Super-Aged Society, Japan: Current Challenges and toward a Research Agenda

**DOI:** 10.3390/ijerph15092054

**Published:** 2018-09-19

**Authors:** Mohammad Javad Koohsari, Tomoki Nakaya, Koichiro Oka

**Affiliations:** 1 Faculty of Sport Sciences, Waseda University, Saitama 359-1192, Japan; koka@waseda.jp; 2 Behavioural Epidemiology Laboratory, Baker Heart and Diabetes Institute, Melbourne 3004, Australia; 3 Mary MacKillop Institute for Health Research, Australian Catholic University, Melbourne 3000, Australia; 4 Graduate School of Environmental Studies, Tohoku University, Sendai City 980-0845, Japan; tomoki.nakaya.c8@tohoku.ac.jp

**Keywords:** urban design, active living, aging, physical activity, sedentary behavior, age-friendly environments

## Abstract

There is a growing recognition of the role of built environment attributes, such as streets, shops, greenways, parks, and public transportation stations, in supporting people’s active behaviors. In particular, surrounding built environments may have an important role in supporting healthy active aging. Nevertheless, little is known about how built environments may influence active lifestyles in “super-aged societies”. More robust evidence-based research is needed to identify how *where* people live influences their active behaviors, and how to build beneficial space in the context of super-aged societies. This evidence will also be informative for the broader international context, where having an aging society will be the inevitable future. This commentary sought to move this research agenda forward by identifying key research issues and challenges in examining the role of built environment attributes on active behaviors in Japan, which is experiencing the longest healthy life expectancy, but rapid “super-aging”, with the highest proportion of old adults among its population in the world.

## 1. Built Environments, Physical Inactivity, and Aging

Physical inactivity (defined as lack of exercise and prolonged sitting time) is one of the leading risk factors for most chronic diseases [1,2]. For example, a systematic review using meta-analysis found an inverse relationship between physical activity and type 2 diabetes [3]. Another recent meta-analysis review of 47 studies demonstrated the association between too much sitting time with chronic disease risk, regardless of time spent in physical activity [4]. Nevertheless, the rate of physical inactivity has been constantly increasing across the world over the last decades [5]. For instance, a study using pooled data from 76 countries found one in five adults in the world is physically inactive [6].

Motivating individuals to make lifestyle changes is important in promoting physical activity and reducing sedentary behavior [7]. However, since people’s daily active behaviors are highly habitual (not involving conscious decisions), such interventions targeting individual motivation may not be totally effective [8]. There is a growing recognition of the role of built environment attributes, such as streets, shops, workplaces, greenways, parks, and public transportation stations, in supporting people’s active behaviors [9,10]. For instance, an international study using data from 14 cities worldwide found built environment attributes, including higher residential density, well-connected street network, availability of public transport, and higher number of parks, to be positively associated with adults’ physical activity [11]. The fundamental assumption is that even if the effects of built environment interventions on each person’s behavior is small, the overall population effect can be large, and these effects may be sustained over a relatively long-term period [12].

The world population is aging, with the number of persons aged 60 or over expected to be more than double by 2050, compared to 2017, and the number of super-aged societies has been continually increasing [13]. According to the United Nations, a super-aged society refers to a society where more than 20% of their total population is aged 65 years and older. People’s ability to be active and their mobility gradually declines as they age; because of their declining physical function [14]. Older adults may be interested in doing physical activity, but their physical function may substantially limit their mobility. Therefore, their surrounding immediate environment may play an important role in supporting healthy active aging [15,16]. While few studies exist in other regions [17,18], there are many studies examining associations of environmental attributes with active behaviors conducted in developed societies in New Worlds, such as the United States, Canada, and Australia (which are characterized by a Western colonization history, high car dependency, and large proportions of relatively young immigrants). In particular, little is known about how such attributes may influence active lifestyle in super-aged, non-anglosphere societies, such as Japan and Germany [15,19]. For example, in a recent systematic review of built environment attributes related to older adults’ active travel, only one study (out of 42) was conducted in Japan (and no studies were from Germany) [19]. More robust evidence-based research is needed to identify how *where* people live influences their active behaviors, and how to build beneficial space in the context of super-aged societies. This evidence will also be informative for the broader international context, where having an aging society will be the inevitable future.

This commentary sought to move this research agenda forward by identifying key research issues and challenges in examining the role of built environment attributes on active behaviors in Japan, which is experiencing the longest healthy life expectancy, but rapid “super-aging”, with the highest proportion of old adults among its population in the world.

## 2. Key Issues in Activity-Friendly Built Environment Research in Japan

Although the Japanese are the healthiest population in the world, the rate of physical inactivity is increasing in Japan, following the global trend. For instance, according to a study using a nationally representative sample of Japanese adults, the number of adults’ daily walking steps has gradually decreased since approximately 1998–2000 [20]. Japanese adults also reported the highest amount of sitting time per day among 20 countries [21]. In addition, Japan is already a super-aged society with 26.6% of its total population aged 65 years and over in 2015 [22]. Therefore, promoting physical activity has become one of the major public health targets in Japan. “Health Japan 21”—the national plan for health promotion—emphasizes the importance of environments for supporting an active and healthy lifestyle [23].

Several studies conducted in Asian countries examined how environmental attributes can influence active behaviors [24,25,26]. Similarly, the associations between built environment attributes and active behaviors have been investigated in several previous studies in Japan [27,28,29,30,31,32,33,34]. For instance, several perceived environmental measures, including residential density, access to shops, sidewalk availability, and availability of bike lanes, were found to be positively associated with adults’ physical activity in two areas in Japan [28]. Another study found objective measures of population density and the presence of parks to be positively associated with Japanese older adults’ leisure physical activity [33]. And, a recent Japanese study found that residents who lived in areas with well-connected streets were likely to report more walking and less driving, compared with those who lived in less-connected areas [31]. These studies shed light on better understanding the environmental correlates of active behaviors in Japan. They especially provide preliminary evidence about the importance of perceived neighborhood attributes in supporting an active lifestyle. The identified set of influential environmental attributes are somewhat like those reported in the United States, Canada, and Australia. Nevertheless, there are several issues about activity-friendly built environments in Japan—as a super-aged society—which need to be investigated.

### 2.1. Shrinking Cities: An Active Living Opportunity or a Threat?

The Shrinking Cities International Research Network [35] defined a shrinking city as “a densely populated urban area with a minimum population of 10,000 residents that has faced population losses in large parts for more than two years and is undergoing economic transformations with some symptoms of a structural crisis” [36]. Japan’s population has been continually declining since 2005. Except for a few metropolitan areas (such as Tokyo, Osaka, and Nagoya), many other parts of Japan, especially small towns and rural areas, are experiencing severe shrinking. As an emerging research agenda in urban design and planning, shrinking cities may produce many new challenges [37,38]. In relation to activity-friendly neighborhoods, urban shrinking means that Japanese cities and towns can become far less dense and eventually unable to sustain enough facilities to support daily lives, such as retail stores and public transportation, in the near future. Higher residential density has been consistently found to be associated with people’s active behaviors [11,39]. For example, a Japanese study found the average physical activity levels in Japanese cities to be positively correlated with their urban population density [40]. Therefore, managing urban shrinking can potentially be a future issue for walkability in Japan.

At the same time, urban shrinking may create a new opportunity to reshape built environments as more walkable, since newly available vacant houses and spaces will emerge over the entire city region [41]. A key issue in using these newly available spaces in cities will be “land use mix”, referring to having a variety of destinations such as homes, shops, parks, schools, offices, and train stations within a given area. Previous studies have identified land use mix as one of the main neighborhood walkability features [42,43]. The underlying assumption is that those people who live in an area with a high level of land use mix may have better opportunities to be active within their area. This indicates that encouraging land use mix in neighborhood (re)development plans can be key for supporting an active lifestyle. In the context of the United States, the unwalkable residential suburbs, which are occupied by only single-family houses, emerged through the Euclidian zoning system, which allows exclusively single land use for designated zones. Due to the lack of a rigid regulation scheme, Japanese suburbs have occasionally developed unintended mixed land use of various residential zones with commercial, industrial, and agricultural zones in relatively small areas in the age of urban sprawl. However, uncontrolled mixed land use development is not necessarily beneficial for health. For instance, it may increase noise and crime risks, and the emerged landscape is likely to be chaotic and aesthetically less attractive. How to control suburbanization and how to manage the use of newly available (but sporadically) emerging new spaces in existing built-up areas for creating opportunities that support an active lifestyle is vital for the future of shrinking cities.

In response to urban shrinking, Japanese local and national governments are now encouraging compact city policies. The policy may keep enough population density in residential areas to sustain urban facilities, which are an important element of walkability. It may also reduce car dependency of residents by sustaining public transportation service. Compact city may be a response to urban shrinking in cities where already experiencing shrinking; and it may not be a solution to avoid urban shrinking in other areas. In addition, while compact city policies have been widely advocated across the world, their health effects have not been fully examined [44]. Future research is needed to identify challenges and opportunities raised by urban shrinking in super-aged societies in relation to activity-friendly neighborhoods.

### 2.2. Extreme Levels of Environmental Attributes

**Figure 1:**
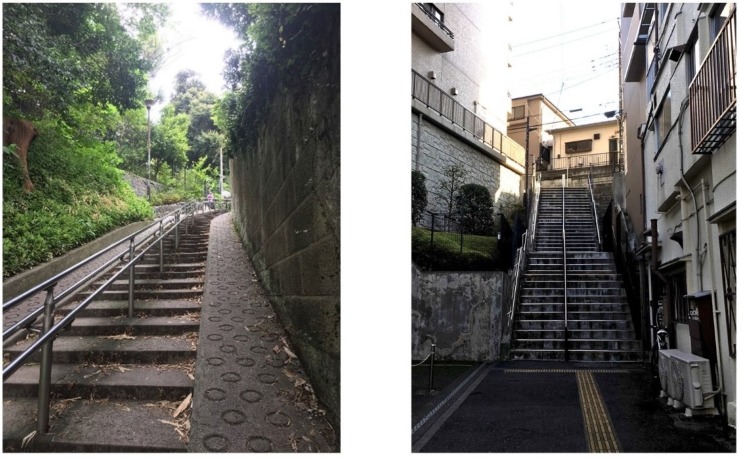
Steep slopes in the central parts of Tokyo, Japan (source: authors).

Apart from land use mix, several differences exist in built environment attributes influencing active life between Japan and Western societies. For example, slope is an environmental attribute that Japanese cities have in extreme levels compared to the United States., Canadian, and Australian cities. About three-quarters of the national land of Japan is mountains, and the residential areas are limited to only four percent of the national land [45]. Slope is one of the typical features of Japanese cities and suburbs. It is not uncommon to see steep slopes (greater than 25 percent), even in central parts of Japanese cities (Figure 1). Some previous studies have shown the positive effects of slope on type 2 diabetes, assumingly through vigorous physical activity [46,47]. However, slope (and subsequently stairs) has been identified as one of the barriers for the elderly to be physically active within their neighborhoods [48,49,50]. Most previous studies examining accessibility measures of built environments, such as access to shops, train stations, and parks, in relation with active behaviors, did not take into account the slope factor [50]. Another extreme level of environmental attribute in Japanese cities is residential density. For instance, the city of Nagoya in Japan has a population density of 7080 persons per square kilometer [51], whereas the population density of Melbourne (the most dense capital city in Australia) is only 450 people per square kilometer [52].

The relationship between environment and active behavior may not be always linear: there may be specific levels (thresholds) over which the effects of a built environment attribute on a behavior may change, especially among the elderly [53,54]. For example, there may be specific amounts of slope or residential density beneficial for elderly active behaviors and health. Nevertheless, most evidence on the importance of built environments on active behaviors comes from Western countries with relatively less extreme levels of environmental attributes [17,18]. This suggests that it is not clear how extreme levels of these environmental attributes may shape elderly’s active behaviors. Investigating the effects of built environments on active behaviors in Japan can provide the international field with an opportunity to identify the optimal values of these environmental attributes for supporting an active aging life.

### 2.3. Exposures to Environments: Time/Place in Active Behaviors

Not only levels but also the way people are exposed to environments or use surrounding environmental opportunities can be distinctively different between societies. In particular, temporality is an important issue in investigating the relationships between environmental attributes and active behavior [55,56]. People are exposed to different types of environments in daily life depending on their mobility status. Consequently, their active behaviors are influenced not only by their immediate residential environment, but also by a broader environmental context. There are several reasons why the temporality in the relationships between environment and behavior may be more important in the context of Japan compared with other regions. First, safety from crime is relatively higher in Japan than other industrialized societies [57]. Since there is a low crime rate even at night-time, time restrictions for walking outside are minimal in Japan. Second, since most convenience stores in Japan are open 24 h a day and 7 days a week, there are still people walking to their local convenience stores even at midnight. Finally, Japan benefits from an efficient public transportation system that includes trains and buses, especially within urban areas. This efficient public transport system enhances the elderly’s mobility and enables them to travel far from their homes (and be exposed to a wide range of environments) in their daily life. Therefore, future studies in Japan need to include time-specific measures of environment and active behaviors.

### 2.4. Health Disparities, Environmental Equity, and Activity-Friendly Urban Design

Reducing health disparities is now a major goal of public health across the world [58]. Health disparities across regions are increasing in Japan. A recent study published in *Lancet* has shown widening life expectancies and clear variations of disease burden across the Japanese prefectures [59]. Larger health disparities may also exist at a smaller areal level in Japan [60]. Several previous studies showed an association between physical activity levels and socioeconomic status (SES): more disadvantaged people are likely to be less physically active [61,62]. For example, a systematic review found people with high SES were more active than those with low SES during leisure time [61]. Mitigating the physical activity gap between low and high SES areas can be an important step in reducing the health disparity, especially among elderly [63]. Inequitable distribution of environmental attributes supporting physical activity (e.g., commercial destinations, parks, and well-connected streets) across low and high SES areas may be one of the reasons for this gap. Several previous studies showed those who lived in more deprived areas have less walkable built environment attributes [64,65]. For instance, a national study conducted in Germany found a significant positive association between income level and the amount of urban green space [65]. However, some studies found either no SES disparities or clear patterns in access to walkable neighborhood attributes [66,67]. For example, a recent national study conducted in the United States found a complex relationship between SES and walkable neighborhood attributes: those who lived in more disadvantaged areas, or in areas with more educated people, had better environmental attributes conducive to walking [66].

Nevertheless, there are few studies yet to investigate whether disadvantaged people have poorer walkable neighborhood attributes in Japan. For example, access to parks was found to be poorer for disadvantaged areas in Yokohama City, Japan, compared with affluent areas [68]. Another recent Japanese study found deprived neighborhoods to be less walkable in terms of population density, street density, and access to commercial concentrations [69]. However, little is known if changes in such environmental attributes could alter the health disparity. Considering the life course epidemiological approach, past environmental disparities may contribute to health disparity in later life [70]. Identifying both historical and geographical patterns between differing SES areas with walkable neighborhood attributes will guide urban design interventions to reduce the physical activity gap between these areas, and ultimately reduce the health disparities across regions.

## 3. Conclusions: Toward a Research Agenda

There has been a growing body of research examining how built environment attributes can influence active behaviors. We have identified key issues and challenges providing robust evidence-based research on the role of the surrounding physical environment on people’s active life in the context of a super-aged society. To summarize, the following issues need to be investigated in future studies:

- explore challenges and opportunities that shrinking cities will have on active behaviors;
- identify optimal levels of environmental attributes, such as residential density and slope, needed to support healthy active aging;
- understand time/place in the elderly’s active behaviors (the way elderly are exposed to environments or use surrounding environmental opportunities); and
- examine disparities in the distribution of activity-friendly environmental attributes.

In this commentary, our focus was only on walking, as the most common type of physical activity, especially among older adults. There are other types of physical activities, such as exercise, which may be of interest for elderly. Understanding how built environments may influence exercise among elderly requires further research. Cross-disciplinary research, between urban design/planning, sport sciences, public health, transport, geography, and gerontology, is needed to build evidence on how to build, retrofit, and sustain activity-friendly built environments in the context of a super-aged society.
